# Non-Resorbable Nanocomposite Membranes for Guided Bone Regeneration Based on Polysulfone-Quartz Fiber Grafted with Nano-TiO_2_

**DOI:** 10.3390/nano9070985

**Published:** 2019-07-08

**Authors:** Gheorghe Nechifor, Eugenia Eftimie Totu, Aurelia Cristina Nechifor, Ibrahim Isildak, Ovidiu Oprea, Corina Marilena Cristache

**Affiliations:** 1 Department of Analytical Chemistry and Environment Engineering, Faculty of Applied Chemistry and Materials Science, University Politehnica of Bucharest, 1-7 Polizu St., sector 1, 011061 Bucharest, Romania; 2 Department of Bioengineering, Faculty of Chemical and Metallurgical Engineering, Yildiz Technical University, 34210 Esenler-Istanbul, Turkey; 3 Department of Inorganic Chemistry, Physical Chemistry and Electrochemistry, Faculty of Applied Chemistry and Materials Science, University Politehnica of Bucharest, 1-7 Polizu St., sector 1, 011061 Bucharest, Romania; 4 Department of Dental Techniques, Faculty of Midwifery and Medical Assisting (FMAM), “Carol Davila” University of Medicine and Pharmacy, 8, Eroilor Sanitari Blvd, 050474 Bucharest, Romania

**Keywords:** silica microfiber, nanotitania, polysulfone nanocomposite membranes, chemical oxidation resistance, guided bone regeneration

## Abstract

The polymer-inorganic nanoparticles composite membranes are the latest solutions for multiple physicochemical resistance and selectivity requirements of membrane processes. This paper presents the production of polysulfone-silica microfiber grafted with titanium dioxide nanoparticles (PSf-SiO_2_-TiO_2_) composite membranes. Silica microfiber of length 150–200 μm and diameter 12–15 μm were grafted with titanium dioxide nanoparticles, which aggregated as microspheres of 1–3 μm, applying the sol-gel method. The SiO_2_ microfibers grafted with nano-TiO_2_ were used to prepare 12% polysulfone-based nanocomposite membranes in N-methyl pyrrolidone through the inversion phase method by evaporation. The obtained nanocomposite membranes, PSf-SiO_2_-TiO_2_, have flux characteristics, retention, mechanical characteristics, and chemical oxidation resistance superior to both the polysulfone integral polymer membranes and the PSf-SiO_2_ composite membranes. The antimicrobial tests highlighted the inhibitory effect of the PSf-SiO_2_-TiO_2_ composite membranes on five Gram (-) microorganisms and did not allow the proliferation of *Candida albicans* strain, proving that they are suitable for usage in the oral environment. The designed membrane met the required characteristics for application as a functional barrier in guided bone regeneration.

## 1. Introduction

Guide bone regeneration (GBR) is a standard procedure applied for oral bone defects reconstruction in order to replace the missing hard tissue lost due to periodontal diseases, tooth extraction, trauma, and tumors. The barrier membrane, with the essential role of preventing ingrowth of the non-osteogenic tissues in the bone defect site, is a crucial component of the treatment’s success [1,2]. The requirements for an ideal membrane indicated for GBR are tissue adhesion without mobility, blocking of soft tissue in-growth, easy use, maintenance of space, biocompatibility, and antibacterial properties [2,3,4]. Despite the concept, according to, the membrane is a passive barrier for soft-tissue invasion. Ideally, it should also directly promote the sequences of biological processes leading to bone regeneration and filling the defect with mature, remodeled bone [2]. Various non-resorbable (polytetrafluoroethylene-PTFE or titanium mesh) and resorbable (collagen; PCL—poly(ε-caprolactone); PGA—poly(glycolic acid); PLA—poly(lactic acid)) membranes were proposed as barriers for GBR, but none satisfy all the required properties [2,3,4]. Most of the non-resorbable membranes had a significant complication associated to vertical ridge augmentation, lack of soft tissue, and the exposure in the oral environment and subsequent bacterial colonization, necessitating premature retrieval of the membranes and bone graft loss. On the other hand, for resorbable membranes, resorption time needs to be strictly controlled and longer than new bone formation time; the effect of their degradation, mostly via hydrolysis, creates an acid environment, which may hurt hard tissue regeneration [3].

Membranes and membrane processes constitute the high technology field in which polymers have capitalized on their entire mechanical, thermal, chemical and economical potential. Starting from biopolymers and artificial polymers (cellulose and derivatives) [5,6,7], the membranes were able to develop through technopolymers (polysulfones [8], polyamides [9], polyimides [10], polyetherketones [11]) with a higher physicochemical resistance, especially to oxidative degradation and biodegradation [8,9,10,11]. At the same time, special polymers (polyoxazoles [12], polylactones and polylactams [13,14,15], fluorinated polymers [16], and polycrownethers [17]) contributed decisively to the development of the most advanced membranes in industrial processes: Gas separation, pervaporation, pertraction, ion exchange, chiral resolution, and combustion cells.

While the integral polymer membranes (symmetric or asymmetric) allowed the passage of the membrane processes from the laboratory scale to industrial implementation (the production of drinking and ultrapure water, chemistry and petrochemistry, food industry, hydrometallurgy), they fail to meet all the current requirements of biomedicine, biotechnology, microelectronics, energy production (fuel cells), or ecological technologies [18].

The increasingly sophisticated membrane requirements, such as chemical and photochemical oxidation resistance, high selectivity, sensitivity to physical, chemical and biological parameters, high flow at low pressures, and extended lifetime, have to lead to the transition from classical membranes to composite membranes [19,20].

The success of the composite membranes has been emphasized by the development of micro and nano chemical species’ synthesis: Carbon nanotubes [21], fullerenes [22], graphene [23], and metallic and nonmetallic oxides [24,25]. Nevertheless, the broadest applicability is related to the various forms of silica that have been used [26,27,28,29].

Despite the registered progress in membrane functionalization, biocompatibility is of high interest when the polymeric membrane is intended for usage in the oral environment.

Polysulfones, a class of amorphous thermoplastic polymers, were proposed as orthopedic and implant material, with promising results in animal models [30,31], for alveolar ridge augmentation in conjunction with a demineralized bone powder [32] or as denture base reinforcement [33]. Titanium oxide presents excellent biocompatibility [34] and recently proved positive influence in osteoblast adhesion and differentiation [35,36]. The concept behind the choice of SiO_2_ was due to the biocompatibility of silicate-based glasses. Moreover, Aldaadaa and coworkers proposed a synthetic bone graft material, phosphate glass, dopped with 5 mol% SiO_2_ and 5 mol% TiO_2_ with improved biological responses and controllable degradation rate [37] for bone tissue engineering.

It is also known that the nanosized titania particle coating on the prosthetic implants is used for controlling post-operative complications and infections, due to its antimicrobial action [38]. Also, nano-TiO_2_ has a photocatalytic effect, as, under UV illumination, it generates reactive oxygen species, which can oxidize membrane lipids, finally causing the death of bacterial cells [39]. Due to the biocompatibility feature of nano-TiO_2_, this material is widely used in pharmaceutical and clinical applications.

In the context of high interest for oral environment GBR, the present paper addresses the obtaining, complex characterization, and membrane process characterization of new polysulfone-silica microfibers—nano titanium dioxide (PSf/SiO_2_ + TiO_2_) nanocomposite membranes. The newly obtained membrane system attempts to combine the performances of the polymer-silica and polymer-titanium dioxide nanocomposites, able to better fulfill the requirements for GBR regarding biocompatibility, porosity, and mechanical and chemical resistance, in the meantime exhibiting antibacterial properties.

## 2. Materials and Methods 

### 2.1. Materials and Equipment 

The polysulfone (PSf) used, type Udel (Solvay Bucharest SRL, Romania), had medium molecular mass (28,000 D). Other chemicals used: Bovine serum albumin (BSA—MW 67,000 D), sulphuric acid, sodium hypochlorite, N-methyl pyrrolidone (NMP), and ethylic alcohol (A) were purchased from Sigma Aldrich (Sigma-Aldrich, Merck KGaA, Darmstadt, Germany). Also, the t-butyl alcohol, potassium t-butoxide, and tetra t-butylate titanium were from Sigma Aldrich (Sigma-Aldrich, Merck KGaA, Darmstadt, Germany). The silica microfibers (Lanxess NV, Antwerp, Belgium) resulted from the processing of the optical fibers, and microfiltration membranes’ fibrils (MerckMillipore, Merck KGaA, Darmstadt, Germany) were used in experiments. The tubular cellulosic membrane type was MEDICELL International Ltd. (Dialysis Tubing-Visking). A Millipore System (MerckMillipore, Merck KGaA, Darmstadt, Germany) provided the necessary ultrapure water (W).

For mixing up the chemicals, an ultrasonic bath was used (Elmasonic S10 H, Elma Schmidbauer GmbH, Singen, Germany). Electrochemical measurements were performed on a conductometer—Cyberscan PCD 6500 (EUTECH Instrument, Thermo Fisher Scientific Inc., Dreieich, Germany) and dielectric analyzer—DEA 288—Netzsch (NETZSCH-Gerätebau GmbH, Selb, Germany). The detection sensor used was Coated Tool Mountable Comb Electrode with 0.5 mm electrode spacing from Netzsch (NETZSCH-Gerätebau GmbH, Selb, Germany). The membrane film thicknesses were approximately 5 mm, and the measurement frequency for each test was 10 Hz. Substances weighted with the aid of an analytical balance type KERN ALT 220-4NM (Merck KGaA, Darmstadt, Germany) and the samples were dried using a drying cabinet with air flow (Memmert GmbH, Schwabach, Germany). 

The morphology studies were done through SEM analysis equipped with EDX facilities (FESEM Hitachi S4500- Hitachi High-Technologies Europe GmbH, Krefeld, Germany).

The thermal properties of the obtained composite membranes were analyzed using a Netzsch Thermal Analyzer (NETZSCH-Gerätebau GmbH, Selb, Germany). Measurements for the samples placed inside an aluminum pan were done under nitrogen atmosphere. The applied heating rate was 10 °C/min for a temperature ranging from ambient to 1000 °C, in order to follow up the thermal effects during the heating processing.

### 2.2. Procedure for Preparing Quartz Fibers Grafted with TiO_2_

Into a 250 cm^3^ three-necked flask equipped with a mechanical stirrer and a reflux refrigerant, 150 cm^3^ of t-butyl alcohol and 5 g of cut optical fibers (quartz powder) were introduced under stirring. After the quartz particles became transparent, we added 2.5 g of potassium t-butoxide, and the mixture was refluxed for 30 min under continuous stirring to activate the surface of the silica fibers. The mixture homogenization continued for 4 h after stopping the heating. The reaction mixture was filtered on a Sartorius microfiltration installation (Sartorius GmbH, Goettingen, Germany) and washed 5 times with 100 cm^3^ of t-butyl alcohol for removing the potassium t-butoxide excess.

Afterward, 50 cm^3^ of t-butyl alcohol and 0.5 g of titanium tetra t-butylate were introduced into a 100 cm^3^ conical flask with a pennyhead stopper, and the vessel was immersed for homogenization in an ultrasonic bath for 1 h. In order to suspend the microfibers and complete the TiO_2_ gelling reaction onto their surface, we introduced 1 g of silica microfibers, and the homogenization was continued for 3 h. The final reaction mixture was separated by microfiltration. Finally, the obtained microfibers grafted with titanium dioxide were ready for SEM and EDX characterization.

### 2.3. Obtaining the Composite Membranes PSf-Quartz Fibers

The 12% (*w*/*w* %) solution of PSf in NMP was obtained by dissolving 12 g of polymer and 88 g of solvent (97 cm^3^, ρ = 1.028 g/cm^3^) in a conical flask equipped with a magnetic stirrer. The polymer dissolved in the solvent when gradually added under continuous stirring. After 4 h, the solution was divided into 4 vials, and 0.30 g of silica microfibers and membrane fibrils were added under ultrasonication and mixed for two hours. Then, the solutions were left at rest for gas bubbles release. Subsequently, they were cast on optical glass plates, into a 500 μm film, and left in the open air at room temperature for evaporation. Finally, they were immersed for coagulation into the water:ethanol (1:1 ratio) mixture for 24 h. The obtained membranes were washed 5 times with 50 cm^3^ mixture ethyl alcohol:ultrapure water (1:1 ratio) and then stored in ultrapure water cassettes.

### 2.4. Obtaining the Composite Membranes PSf-Quartz Fibers Grafted with TiO_2_

Applying a similar procedure as described earlier (Section 2.3), a new 12% PSf/NMP solution was obtained. This time, after 4 h of mixing, the solution was divided into 5 vials, and under ultrasonication 0.30 g of the silica microfibers grafted with TiO_2_ were added. The new solution was homogenized for two hours and then cast onto optical glass plates as 500 μm thin film. The polysulfone-silica-nano titania (PSf-SiO_2_-TiO_2_) composite membranes were obtained through solution coagulation in the presence of ultrapure water:ethanol (1:1 ratio) mixture. These composite membranes were washed 5 times with 50 cm^3^ of ultrapure water. They were stored wet in cassettes. 

### 2.5. Membranes’ Characterization

#### 2.5.1. Gravimetric Determination of the Total Porosity 

**The porosity** (*ε*) represents the voids fraction of the membrane structure, and represents the ratio between the volume of all the pores and the apparent volume of the membrane:

(1) $$\varepsilon =\frac{\left(V-V’\right)}{V}$$

where:

*V* = apparent volume of the membrane (cm^3^);

*V’* = real volume of the membrane (cm^3^).

Two fundamental dimensions, flux and selectivity, define the membrane performances in terms of separation capacity and flow characteristics.

#### 2.5.2. Determination of the Process Performances (Flux, Selectivity)

**The flux** (*J*) is generally defined as the fluid volume flowing through the membrane in the unit of time unit per unit area:

(2) $$J=\frac{V}{S\cdot t}$$

where:

*J* = flux (L/m^2^·h);

*V* = volume of fluid flowing through the membrane (L);

*S* = surface area of the membrane (m^2^);

*t* = time (h).

The membranes cut as disks with a diameter of 49 mm and the active filtering surface in Sartorius funnel was 10 cm^3^. In particular, for a comparative study of the membranes, both the flow of solvent (water or ethanol) and solute (BSA), expressed as volume or mass of these related to surface area and time should be determined.

The decision to use a membrane in a specific process is primarily based on a standardized feature called “normalized flow of distilled water,” which is the volume of distilled water flowing through a membrane over a specific time at a given pressure difference:

(3) $$J=\frac{V}{S\cdot t\cdot \Delta p}$$

where:

*J* = flux (L/m^2^·h·bar);

*V* = volume of distilled water (L);

*S* = surface area of the membrane (m^2^);

*t* = time (h);

Δ*p* = pressure difference (bar).

**The retention or selectivity** of the membranes represents their ability to retain on their surface a single component from a complex mixture; in our case, BSA from an aqueous 1 g/L solution. It represents the degree of retention (*R*), determined according to the formula:

(4) $$R=\frac{c_{f}-c_{p}}{c_{f}}=1-\frac{c_{p}}{c_{f}}$$

where:

*c_f_* = solute concentration from the feeding fluid (% mass, g/L, or mol/L);

*c_p_* = solute concentration in permeate (% mass, g/L, or mol/L).

#### 2.5.3. Surface Morphology Characterization 

Membrane samples (1 cm × 1 cm) were mounted on aluminum specimen support and introduced into the electron microscope system—FESEM Hitachi S4500 (Hitachi High-Technologies Europe GmbH, Krefeld, Germany). For high resolutions, the samples were covered with gold, but for combined SEM–EDX analysis, this procedure was avoided. The electronic microscopy offers the possibility of visualizing the porous structure of the membranes. Scanning electronic microscopy, through the analysis of the top surface and the cross-section, gave a clear picture of the overall membrane structure. Thus, the thickness of the active and macroporous layer could be determined, and it was possible to estimate the porosity and pore distribution. 

#### 2.5.4. Determination of Resistance to Oxidizing Agents

Five dry samples of composite membranes (1 cm × 1 cm) were weighed individually and then placed for 48 h in a 200 cm^3^ conical vial with a rounded plug containing 100 cm^3^ of the test oxidation solution (3% hydrogen peroxide in 2% sulfuric acid or hypochlorite sodium 5%). After 48 h, the membranes were removed and placed in a dialysis bag (cellulosic tubular membrane, MEDICELL Membranes Ltd., Greenwich, UK) and washed with ultrapure water. The dialysis stopped when the wash water had constant conductivity. Then, the membranes were removed from the dialysis bag and dried to constant weight (gravimetric method) in the heating cabinet at 60 °C. 

The following relationship allows the calculation for membrane weight loss (*MWL*):

(5) $$MWL=\frac{m_{0}-m_{i}}{m_{0}}=1-\frac{m_{i}}{m_{0}}$$

where *m*_0_ is the initial mass of membrane (mg) and *m_i_* is the mass of the membrane after corrosion (mg).

### 2.6. Mechanical Tests

The membranes’ thickness measurements were done with a digital caliper Mitutoyo (Mitutoyo, QuantuMike Series 283-IP65, Mitutoyo SRL Romania, Otopeni, Romania) with a resolution of 0.001 mm, with 2 mm/rev spindle feed and carbide measuring faces. Such caliper applied the lowest possible pressure over the extended membrane area; therefore, no significant sample deformation occurred. After thickness measurements, the membrane samples were used for mechanical testing. For mechanical tests, a universal testing equipment type INSTRON (INSTRON 3382, Instron^®^ GmbH, Darmstadt, Germany) with a load of 2 kN was used. The displacement measurement accuracy was 0.02 mm, and the testing speed range was 0.5 mm/min. The tensile stress (MPa) against tensile strain (%) was recorded. For our membranes, the properties determined from the tensile stress were the elongation to break, ultimate tensile stress (MPa), and elasticity modulus. The elasticity modulus was calculated as the medium slope of the first linear portion recorded from the tensile stress–tensile strain curve. The results obtained are presented as an average value with the associated standard and mean deviation. Five samples were analyzed for each type of membrane under study. The mechanical tests were run in dry conditions on rectangular specimens of 10 mm (W) × 50 mm (L). The gauge length was 20 mm (distance between clamps) for each sample. 

The modulus of elasticity, *E,* is a vital material property that describes the material stiffness quantitatively. According to Hook’s Law, stress is directly proportional to strain, according to:

(6) σ=E·ϵ

where ***E*** stands for modulus of elasticity (MPa), *σ* represents stress (MPa), and *ε* is the strain (% or unitless). Therefore, the modulus of elasticity (Young modulus) is given by the ratio between stress and strain according to:

(7) $$E=\frac{\sigma }{\varepsilon }$$

### 2.7. Antimicrobial Assessment for Polysulfone Composite Membranes

The disk-diffusion method allowed the assessing of the antimicrobial activity of the composite membranes. The used bacterial strains and the necessary chemicals for preparation of the microorganism cultures were from “Cantacuzino” Medico-Military Research and Development National Institute, Bucharest, Romania. The diffusion tests were performed on five microorganisms belonging to the diderm group bacteria: Three Gram (-) bacteria, *Escherichia coli* ATCC O_125_, B_15_, *Salmonella* PT 10052, *Shigella Shiga*, and two non-fermentative Gram (-) pathogenic bacteria from the *Pseudomonas* class, *Pseudomonas aeruginosa* ATCC 27853 and *Pseudomonas aeruginosa* ATCC 15442. Using the 24 h microbial cultures obtained on nonselective *Gelose* medium (trypticase soy broth—TSA), suspensions were made in saline peptone water with densities corresponding to 0.5 McFarland. These suspensions were seeded on Mueller-Hinton agar medium (for bacteria) in a 9 cm diameter Petri dish with a sterile inoculated swab. The Petri dishes were kept for 15 min at 35 °C. The disks (d = 6 mm) cut from the composite membranes were introduced in the Petri dishes applying a circular layout at equal distances: 3 cm from disc centers and 1.5 cm from plate edge, being radial placed towards the plate center. After introducing the membrane specimens, the plates were placed at room temperature for 90 min in order to assure the uniform diffusion of the substances in the seeded media. Then, the plates were incubated at 35 °C for 24 h. The results were obtained by measuring the diameters of the inhibition areas, namely, the zone of inhibition (ZOI), generated by the tested membranes. All analyses were performed in triplicate.

In addition, the behavior of the composite materials in the presence of the yeast-type fungi, *Candida albicans* ATCC 10231, which is a known pathogenic yeast [40], was tested. The main stages of the testing procedure, which is similar with the above-introduced method, supposed: the preparation of the fungal dilution (0.5 McFarland) from the *Candida albicans* reference strain; the placement of the membranes in sterile Petri dishes; usage of a Sabouraud glucose agar (SGA) medium plate with the same type of fungal dilution for fungal-growth control; preservation of inoculated samples; incubation for 24 h; and samples reading. Each experimental determination was repeated thrice, and each time the readings were taken on three different directions, and the average values were reported.

## 3. Results

For complying with the requirements of an ideal GBR membrane, a polysulfone-quartz fiber grafted with nano-TiO_2_ was proposed and characterized.

**Figure 1:**
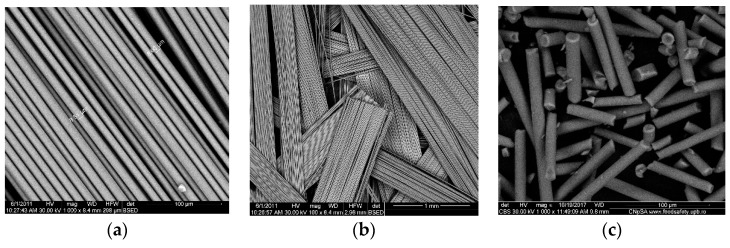
SEM images for: Optical fibers: Fabrics (**a**) and (**b**); optical fiber after cutting (**c**).

Optical fibers and quartz fabrics resulted as waste from the optoelectronics industry represent an excellent source for silica microfiber used in this research, (Figure 1a,b). The microfibers from optical fiber or quartz fabrics have the advantage of being dimensionally homogeneous. The diameters are constant for a particular type of fiber (Figure 1c) and their length is adjusted by an automated cutting installation.

The images from Figure 1 are representative for the silica microfibers homogeneity and quality of the surface. The details shown in Figure 1a,b are relevant and suggestive of the fibers’ diameter and the uniformity of their surface.

**Figure 2:**
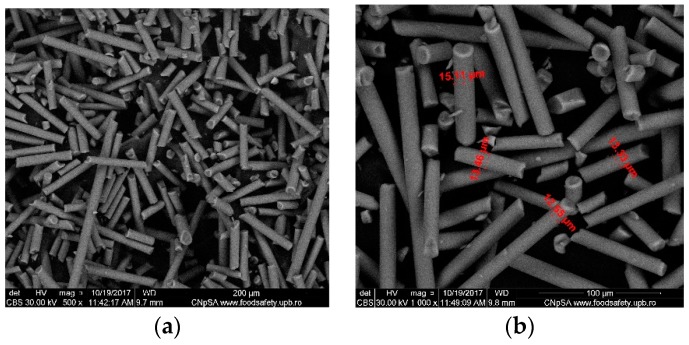
Morphologically characterized optical silica microfiber: Assemblies (**a**) and detail of the fiber diameter (**b**).

Fibrillation leads to relatively monodisperse micro-fibrils, Figure 1c, of about 150–200 μm in length (Figure 2a) and 12–15 μm in diameter as shown and measured in Figure 2b.

All composite membranes obtained in this paper were prepared by phase inversion in two steps:

- Ambiental evaporation of the polymer film for 20 min (membrane preform);
- Precipitation through immersion in a coagulation mixture of equal volumes of water and ethanol for 24 h (maturation of the membrane to fix morphology).

**Figure 3:**
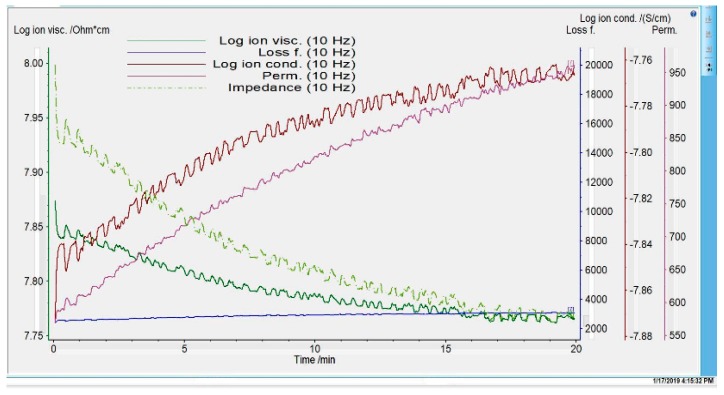
Evolution of electrochemical parameters of polymer film during membranes’ preforming.

The ambient evaporation time was determined by analyzing the evolution of the main electrochemical parameters and loss factor during the formation of the polymeric film from 12% polysulfone solution in N-methyl pyrrolidone (Figure 3).

As seen in Figure 3, the electrochemical parameters of the polymeric film (ionic viscosity, impedance, conductivity, permeability) evolve abruptly during membrane preforming (ambient evaporation) for 15–20 min, after which the values are flattened, which suggests that the membrane preforming has ended.

Table 1 introduces the experimental data recorded for the permeability of polysulfone and polysulfone composite membranes. The increased permeability for ethanol compared to water for all membrane types is easily observed.

According to the procedure presented in Section 2.5.4, the resistance at the oxidizing agents, NaClO and H_2_O_2_, in H_2_SO_4_ was determined. The data in Table 2 present the weight loss (%) of the polysulfone membranes in the presence of oxidizing agents. The parameter values were higher in the presence of NaClO (5%) for polysulfone and polysulfone-SiO_2_ microfiber membranes compared with the values obtained for H_2_O_2_ in the H_2_SO_4_ system. For polysulfone-SiO_2_-TiO_2_ composite membrane, there is a reversed situation, as it recorded a higher weight loss in the presence of H_2_O_2_ in H_2_SO_4_ and not under NaClO action.

Also, the use of TiO_2_ grafted silica fibers increased the retention (selectivity) of bovine serum albumin (MW = 67,000 D) for the obtained nanocomposite membranes (Table 3) significantly. 

The inclusion of silica grafted with nano-titania into a polysulfone matrix is a viable option that facilitates the tailoring of a specific and desired selectivity.

**Figure 4:**
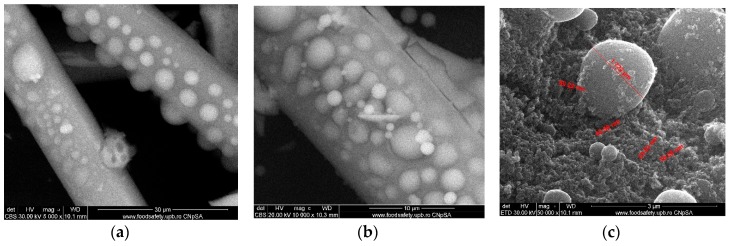
Titanium dioxide grafted silica microfiber after surface activation: Silica microfiber with titanium dioxide (**a**,**b**); detail to highlight the dimensions (**c**).

The quartz microfibers with adherent titanium dioxide, decorating the surface relatively evenly (Figure 4a,b), were obtained after the activation of the quartz microfibers with potassium t-butoxide. Due to the agglomeration of the in-situ formed titania nanoparticles, the grafted titanium dioxide particles present a spherical shape with 1–3 μm diameter, as presented in Figure 4c.

**Figure 5:**
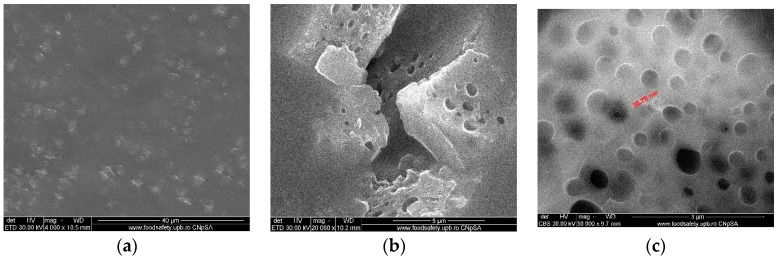
Polysulfone-titanium dioxide grafted silica microfiber composite membranes. (**a**) Surface, (**b**) section, and (**c**) pores’ detail.

The titanium dioxide polysulfone-microfiber (PSf/SiO_2_ + TiO_2_) composite membranes have the morphology evidenced by SEM images presented in Figure 5a–c. The membranes’ surface is uniform and penetrated by microfibrils, Figure 5a, and the sponge-like structure of the cross-section is specific to the membranes obtained by evaporation, Figure 5b. Figure 5c highlights the nano-dimension of pores.

**Figure 6:**
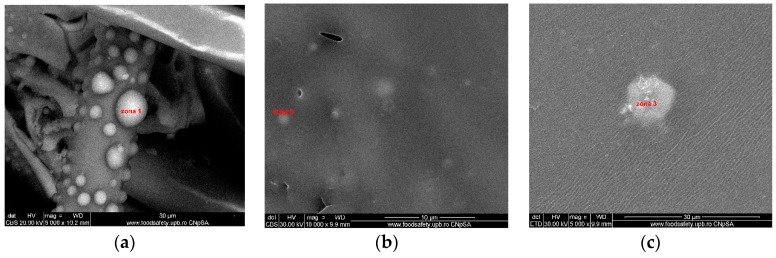
SEM images of microfiber composite membranes: (**a**) Surface of titanium dioxide spherical decoration, (**b**) microfiber surface, and (**c**) top surface of the membrane.

The EDX analysis highlighted the presence of C, S, and O in the polysulfone composition and Si and O for microfibers, while Ti and O were identified for the grafting particles. The investigations were performed in specific areas of the composite membrane: titanium dioxide microspheres, Figure 6a; sections from the membrane’s top surface, Figure 6b,c. 

**Figure 7:**
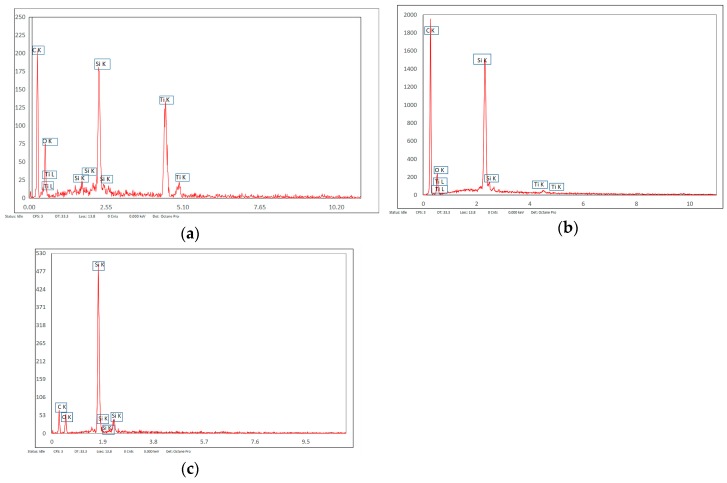
EDX analysis—surface composition of polysulfone—titanium dioxide grafted silica microfiber composite membranes: (**a**) Surface of titanium dioxide spherical deposition, (**b**) silica microfiber grafted with titania, and (**c**) top surface of the composite membrane.

The results indicate a predominance of titanium dioxide microspheres, which resulted from the agglomeration of the TiO_2_ nanoparticles, on the membrane microfibers, Figure 7a. Also, a predominantly titanium-based composition on the surface of the microfibers embedded in the polymer, Figure 7b, was put in evidence. The composite membrane top surface is characterized by the presence of carbon, sulfur, oxygen, silicon, and different titanium contents, Figure 7c.

**Figure 8:**
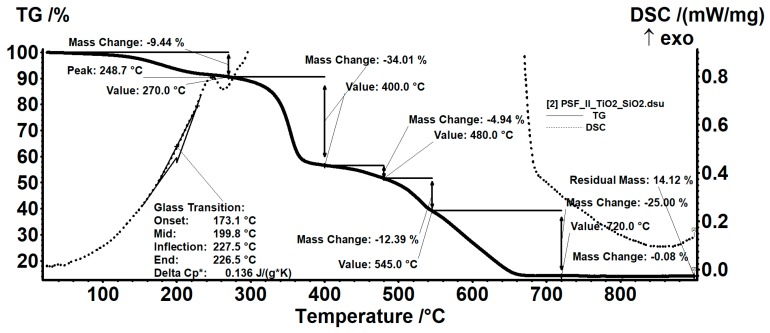
TGA curve for polysulfone-SiO_2_-TiO_2_ composite membrane.

The stability and specific thermal behavior of the nanocomposite polysulfone membrane are presented in Figure 8. Up to 120 °C, the composite membrane loses 1.37% of its mass, most likely due to the evaporation of the remaining solvent and possibly absorbed water, so we can consider that the membranes do not decompose up to this temperature [41]. The principal thermal decomposition occurs from 270 °C on when oxidative thermal processes are present. The formation of the final stable form—the metallic oxides— occurs at temperatures over 720 °C. 

The tensile testing permitted to assess the membrane’s mechanical behavior under a controlled tension/force until its failure. The resulting stress was measured when longitudinal strain acted at a constant rate along with the sample size. In Table 4 are presented the recorded results for the mechanical tests. As observed, there was a significant difference between the thickness of polysulfone membranes, with an average value of 0.426 mm, and the composite membranes.

From Table 4 data, it results that the tensile tests are similar for all membrane samples studied, although there are some limited differences. In Table 5 are introduced the statistical parameters calculated for the mechanical properties of the studied membranes. 

**Figure 9:**
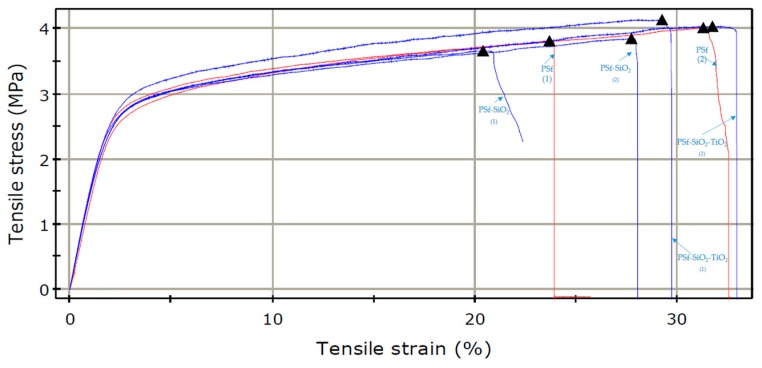
Strain-stress curves for polysulfone-based membranes.

Figure 9 depicts the stress-strain curves recorded for the polysulfone-based membranes. The mechanical tests, along with two orthogonal directions, could evidence the samples’ anisotropy. Anisotropy could affect material behavior under mechanical stress. As observed in Figure 9, for each membrane type, there were recorded two curves. These correspond to the two different samples of each membrane considered for the test: One sample cut along the coagulation direction and the other sample cut across/perpendicular to the coagulation direction of the membranes.

The orthogonal direction used to generate the specimens for tests influenced their response, as shown in the curves in Figure 9. 

Table 6 presents the antibacterial assessment results obtained by applying the qualitative disk-diffusion method. 

The antimicrobial tests performed against the active substrates highlighted the zone of inhibition (ZOI), where bacteria colonies did not grow. For the polysulfone-SiO_2_ microfiber-nano-TiO_2_ membranes ZOI increased.

**Figure 10:**
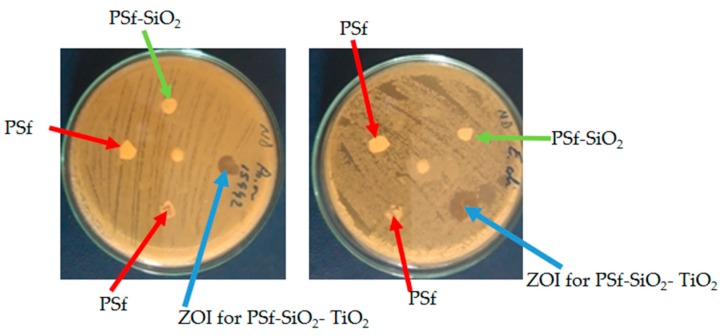
Petri-dish samples showing the presence and absence of ZOI for polysulfone (PSf) composite membranes.

Figure 10 shows the absence of ZOI for PSf and PSf-SiO_2_ membranes when tested for two of the considered microorganisms. 

Table 7 presents the results of the bacteriological analysis against *Candida albicans* ATCC 10231 strain.

**Figure 11:**
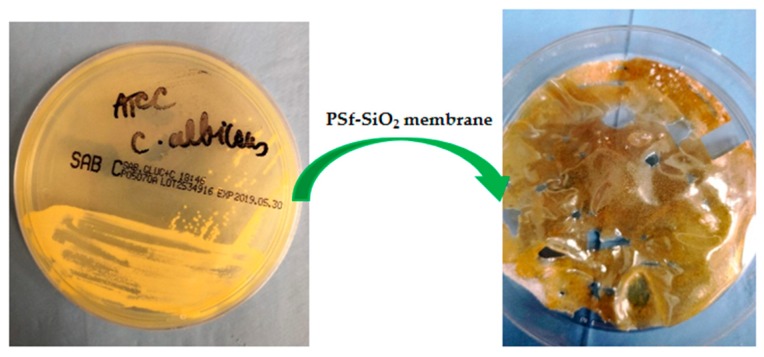
*Candida albicans* proliferation for the tested PSf-SiO_2_ membrane.

The images from Figure 11 evidence the proliferation of the *Candida albicans* strain for the tested PSf-SiO_2_ membrane. Both polysulfone and the PSf-SiO_2_-TiO_2_ membrane did not permit the proliferation of the *Candida albicans* strain, proving that they are suitable for usage in the oral environment. 

## 4. Discussion

The intensive use of polysulfones for the preparation of microporous, dense, and composite membranes is justified by the performance of these polymers [42,43,44], namely: high solubility in polar aprotic solvents and thus great possibilities to obtain membranes by phase inversion; thermal resistance up to approximately 200 °C; chemical resistance over the whole pH range and in oxidative environments; diffusion coefficient of 3.3 × 10^−11^ cm^2^/s at 15 °C; mechanical resistance; and selectivity for gas mixtures. Performance enhancement and widening of polysulfone membrane applications have been amplified by including silica for obtaining micro- or nano-metric silica composites, but their synthesis is often costly, complicated, and non-reproducible [26,45,46,47,48].

The reason to use the glass microfibrils is due to numerous and successful researches for obtaining polysulfone-silica composite membranes, as well as to the fact that they are excellent support for photocatalytic or antibacterial materials, like titanium dioxide particles [49,50,51,52,53].

Nano-titania (TiO_2_), either as filler or as coating, leads to the formation of composite materials that, during recent years, have received considerable attention for applications in the biomedical area due to the excellent biocompatibility. The nano-TiO_2_ particles proved to be safer and to enhance the osteoblast adhesion [54]. It is also known that the nanosized titania particle coating on the prosthetic implants is used for controlling post-operative complications and infections due to its antimicrobial action [38]. Furthermore, nano-TiO_2_ has a photocatalytic effect, as under UV illumination, as it generates reactive oxygen species, which can oxidize membrane lipids, finally causing the death of bacterial cells [39]. Due to the biocompatibility feature of nano-TiO_2_, this is widely used in pharmaceutical and clinical applications. The nano-TiO_2_ insertion in PSf matrix brings higher hydrophilicity, thus preventing the biofilm formation/fooling, in consequence being a viable candidate for clinical applications. A wide range of surface modification techniques for including the nano-TiO_2_ has been introduced lately. The polysulfone-inorganic (SiO_2_) system with nano-titania generates a composite material that provides improved antibacterial behavior and mechanical performance. 

In the present research paper, the used silica microfibers were of length 150–200 μm and diameter 12–15 μm. The morphological studies evidenced their homogeneity and uniform dimensions.

The electrochemical parameters, namely the loss factor and ion viscosity (Figure 3), depict the evaporation progress of the composite membrane at room temperature in an open atmosphere. The lowest viscosity value is reached at 15 min when the evaporation progress ends. The ion viscosity slope shows decreasing reactivity during the evaporation progress. The lowest viscosity corresponding to the highest ion conductivity translates into the best flow behavior. 

Improved polymer flexibility and ionic conductivity for composite membranes are obtained through the dispersion of fillers TiO_2_ or SiO_2_ because the available coordination sites increased. Our experimental data support the role of semiconducting fillers (TiO_2_ or SiO_2_) for the ionic conductivity enhancement. In addition, the impedance variation correlates with the ionic conductivity that directly evidences the presence of a higher quantity of free charge carriers [55,56]. The loss tangent curve plotted for 10 Hz frequency does not record a significant variation, suggesting the absence of fast ion dynamics. The evolution of the followed electrochemical parameters showed that after 20 min, the values became almost constant due to the preforming process completion. In consequence, it was decided to immerse the polymeric film in the coagulation bath for maturation after 20 min of evaporation at room temperature.

Polysulfone-silica fiber composite membranes have a water permeability of about 20% higher than the polysulfone membranes, while the ethylene alcohol permeability is 30% greater. The values recorded for PSf/SiO_2_/TiO_2_ composite membranes showed a permeability increase of 45% for water and 76% for ethylene alcohol (Table 1). 

**Figure 12:**
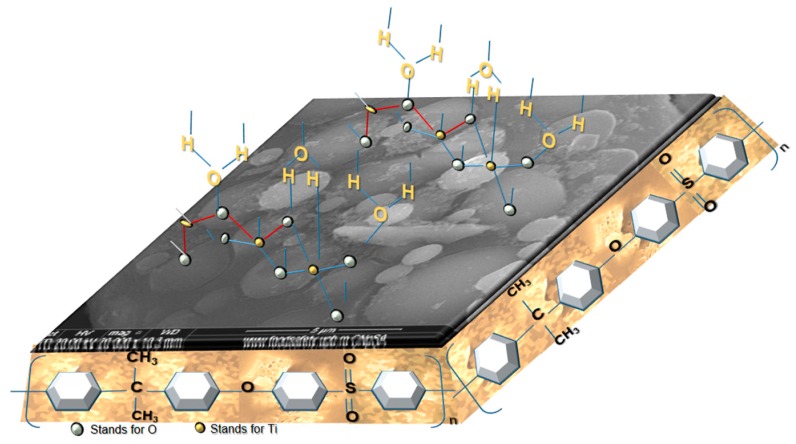
Schematic representation of the polysulfone composite membrane highlighting the hydrophilic surface.

One of the problems encountered when using GBR membranes is the fouling of the bio-compounds from biological fluids, specifically from human saliva. Therefore, it is of high interest to improve the antifouling characteristic of such membranes. It is known that forming a hydrophilic layer onto the membrane surface leads to reduced fouling together with a significant improvement of hemocompatibility of the polymeric membrane [57,58]. Usually, such a hydrophilic surface could be obtained by introducing poly(vinyl pyrrolidone), poly(ethylene glycol), or polyzwitterions [59,60,61]. By introducing the TiO_2_ nanoparticles grafted on silica microfibrils immobilized into the polysulfone matrix, a hydrophilic layer is created—Figure 12—thus enhancing the hydrophilicity of the nanocomposite membrane [62].

Besides, the use of titania nanoparticles in polysulfone-silica microfiber composite membranes provides 39 to 67% more chemical resistance to acid oxidizing agents (3% hydrogen peroxide in 2% sulfuric acid) and alkali (5% sodium hypochlorite)—see Table 2.

For improving the overall performance of polysulfone membranes, silica-titanium dioxide composite microfibers were used for new composite membranes. Thus, a 94% (±5) retention of bovine serum albumin for PSf/SiO_2_ + TiO_2_ composite membranes suggested that both membrane separation and membrane protein interaction increased due to titanium dioxide component inserted into the polymeric matrix (Table 3).

The morphologies of the raw materials—silica microfibers, as well as intermediates—silica microfibers grafted with TiO_2_, and composite membranes, were observed by SEM—Figure 2, Figure 4 and Figure 5. In Figure 2, the images highlighted the smoothness of the exposed surfaces of silica microfibers (raw material). 

The three-dimensional decorating structures presented in Figure 4 are attributed to the adherent deposition of TiO_2_ nanoparticles, which are uniformly dispersed over the silica microfiber surface. However, micro-dimension titania particles have been put in evidence due to the agglomeration of titanium oxide nanoparticles (Figure 4c). Figure 4 shows a large specific area due to the titania particles’ deposition onto the silica microfibrils’ surface. The SEM images of the nanocomposite membranes’ surface shown in Figure 5 point out the uniform distribution of nano-titania grafted silica microfibrils into the polysulfone matrix. The further performed EDX measurements determined the distribution and the existence of SiO_2_ and TiO_2_ inside the membranes—Figure 6 and Figure 7. The characteristic peaks of the elements Si and Ti indicate the homogeneous integration of the grafted silica microfibrils into polysulfone membrane—Figure 7c. 

The data obtained for BSA retention onto Polysulfone composite membranes—Table 3—show that the PSf/SiO_2_ + TiO_2_ composite membranes can retain a larger quantity of protein compared with the simple polysulfone or PSf-SiO_2_ membrane. The experimental data presented in Table 1, Table 2 and Table 3 proves that the polysulfone composite membranes containing SiO_2_ grafted with TiO_2_ have superior properties in terms of flow, retention, and resistance to oxidation compared to PSf or even PSf/SiO_2_ membrane. Such performance parameters offer support for promoting the new composite membrane as a non-resorbable barrier in GBR. Its characteristics would allow washing and sterilization using both acidic and basic (sodium hypochlorite) solutions.

The performed thermal analysis on the nanocomposite PSf membranes showed a 9.44% mass loss in the range of 25–270 °C. A slight exothermic effect accompanies the thermal process in the last part of the temperature range with a maximum recorded at 248.7 °C. The mass loss is due to the removal of remaining solvent embedded in the sample and the removal of the -OH groups from the nanoparticles’ surface as well as to the thermal decomposition of traces from the precursors used in synthesis. The glass transition temperature for the polysulfone composite membrane was 199.8 °C. 

The thermal oxidative degradation that occurred between 270 and 400 °C resulted in a mass loss of 34.01%. A broad, exothermic effect accompanied this process with several peaks (at 356 °C and 384.5 °C, separately), indicating a succession of partially overlapping reactions. There are two other steps with 4.94% loss in the 400–480 °C range and 12.39% mass decrease between 480 and 545 °C. The burning of the residue occurs during the last thermal decomposition stage from 545 °C to 720 °C. This process is accompanied by a strong, broad, asymmetric exothermic effect, with a maximum at 579.9 °C. The final remaining mass is 14.12%, corresponding to the inorganic residue (metallic oxides). The polysulfone-SiO_2_-TiO_2_ nanocomposite membrane proved high thermal stability.

Any mechanical action generates energy that is stored by the material, as reflected in the stress-strain curves. Such curves follow the variation of stress that is the force per unit area and strain, which represents the elongation/contraction per length unit. From the experimental data presented in Table 4, it could be observed that the thinnest membrane was obtained from composite polysulfone-silica microfiber-nano titania, having an average value of 0.207 mm. The statistical parameters associated with the determined mechanical properties for the investigated membranes can be followed in Table 5. The coefficient of variation showed that the dispersion of the values was the lowest for the polysulfone-silica microfiber composite, namely 2.4%.

Nevertheless, the dispersion values are low for the other membranes studied, showing a convergence of the determined values. The absence of discrepancies between the measured thickness values supports the homogeneity of the prepared membranes. Since the thickness consistency could create difficulties regarding the choice of a particular membrane for a specific clinical purpose, this physical parameter should be determined accurately. 

From the Table 4 data, it results that, for the membrane based on the polysulfone-silica microfiber-nano titania composite, the values of the mechanical parameters were slightly better. The values higher by 13.88% for tensile strain and 14% for elastic modulus compared with the polysulfone membrane show improvement, although not too large. According to previous studies, the maximum force acting in an oral environment could range between 148.73 N/cm^2^ (1.48 MPa) and 354.014 N/cm^2^ (3.54 MPa) [63]. In this context, we could consider that our proposed composite membrane polysulfone-silica microfiber-nano titania corresponds from a mechanical properties point of view, as its maximum tensile stress was 4.1 (±1) MPa.

The curves presented in Figure 9 allow to conclude that the sample orientation has a definite effect on the overall mechanical behavior, the samples obtained by cutting the membranes along the coagulation direction (curves (2)) present a better performance compared with the samples that were cut perpendicular to the coagulation direction (curves (1)). The values of the tensile stress and elongation at break (%) for the composite membranes presented a slight increased in the sequence PSf < PSf-SiO_2_ < PSf-SiO_2_-TiO_2_, according to the values in Table 4 associated with the statistic parameters, Table 5. The observed improvement of the mechanical characteristic for PSf-SiO_2_-TiO_2_ could be assigned to the presence of TiO_2_ and the uniformity of the filler introduced in the PSf matrix. 

From the data recorded in Table 6, the absence of an inhibiting effect on the growth of *Escherichia coli, Salmonella, Shigella,* and *Pseudomonas aeruginosa* strains for the polysulfone, and polysulfone-SiO_2_ microfibers membranes is easily noticed. The images from Figure 10 put in evidence the presence of ZOI for the PSf-SiO_2_-TiO_2_ membrane for the *Pseudomonas aeruginosa* ATCC 15442 and *Escherichia coli* ATCC O_125_, B_15_.

The sample from the polysulfone-SiO_2_ microfiber-nano-TiO_2_ membrane proved there is an inhibitory effect for all strains tested revealing zones of inhibition (ZOI) between 10 mm and 16 mm, as presented in Table 6. While not too large, the presence of ZOI when the polysulfone-SiO_2_ microfiber-nano-TiO_2_ was tested encourages the possibility for further usage of this composite in the clinical area, namely for GBR applications. The bacteriological analysis reflected in the results from Table 7 highlights that PSf and PSf-SiO_2_-TiO_2_ membranes did not favor the *Candida albicans* strain proliferation. In contrast with these membranes’ behavior, the composite PSf-SiO_2_ allowed yeast proliferation, see Figure 11. The obtained results support the introduction of nanoparticles of titanium oxide into the PSf matrix for improving the antibacterial action. 

Even though synthetic polymers are extensively used either as non-resorbable or resorbable barrier membranes, to our knowledge, this is the first polysulfone membrane prepared and proposed for usage as GBR. 

Due to its mechanical resistance in reduced thickness, the proposed membrane offers excellent space-maintaining properties for graft material, being sufficiently rigid to withstand the compression of the overlying soft tissue, without the risk of collapsing, especially when vertical bone augmentation is required. The modified polysulfone membrane possesses a degree of plasticity, being comfortably contoured, and molds to the shape of the defect, making it ideal for the GBR technique.

Porosity is an essential property of a GBR membrane; a significant pore size determines the invasion of soft-tissue cells through the membrane, but, on the contrary, a total occlusive barrier prevents the diffusion of fluids, oxygen, nutrients, and bioactive substances required for cell growth and vitality, impairing bone and soft-tissue regeneration [64]. The presence of pores with size 5–30 µm in the PTFE membranes was proved to facilitate bacterial contamination and attachment of soft tissue [65]. Therefore, a submicron (0.2 µm) pore size was developed to avoid the migration of bacteria into the membrane structure [66]. However, the slow rate of bone formation was registered in association with an occlusive barrier [67], and several authors indicated 100–300 µm pores for facilitating bone augmentation [68,69]. The nanocomposite membrane polysulfone-SiO_2_-TiO_2_, characterized in our work, presents a balanced structure-porosity, supporting its further application as a barrier membrane for clinical usage. Also, by including TiO_2_ with proved antibacterial properties [70,71], our proposed membranes are expected to possess antibacterial activity and protect bone graft material against contamination from the oral environment when accidentally exposed.

The selective permeability of the proposed membrane was designed to facilitate fluid and oxygen diffusion from the highly vascular periosteum to the bone graft material. The thermal resistance of our designed membrane allows sterilization with saturated steam (121–134°C), using an autoclave similar to regular surgical instruments [72].

Due to its characteristics, the obtained polymeric nanocomposite membrane is not just a regular barrier against soft tissue proliferation, but, due to its selective permeability and antibacterial properties, it could also directly promote bone regeneration. However, more in-vitro studies are requested to evaluate and improve its features.

## 5. Conclusions

Further development of membranes and membrane processes are stimulated by the possibility of obtaining novel polymer-nanoparticle composite membranes; in particular, composite polysulfone-inorganic nanoparticles.

We have presented the complete procedure to obtain composite membranes based on polysulfone, and titanium dioxide grafted silica microfibers (PSf/SiO_2_ + TiO_2_). Also, these nanocomposite membranes have explicitly been characterized. The 12% polysulfone nanocomposite membrane with added titanium dioxide grafted onto the silica microfibers presents, from a morphological and structural perspective, the specific characteristics of ultrafiltration membranes. Moreover, such membranes have improved performance compared to the entire polymer membranes or silica microfiber membranes—they have increased porosity, superior retention, and increased resistance—making them ideal candidates to be used for GBR.

The composite membranes tested on standard microorganisms *Escherichia coli* ATCC O_125_, B_15_, *Salmonella* PT 10052, *Shigella Shiga* and two non-fermentative Gram (-) bacteria from the class of *Pseudomonas*, namely *Pseudomonas aeruginosa* ATCC 27853 and *Pseudomonas aeruginosa* ATCC 15442, presented a differentiated effect. Thus, the composite membranes polysulfone-silica microfiber and polysulfone membranes did not have a toxic/inhibitory effect on the tested strains, while the polysulfone-SiO_2_ microfiber-nano-TiO_2_ membrane proved to have an inhibitory effect on all strains tested. The results recorded after 24 h incubation for the *Candida albicans* strain showed that the polysulfone composite with silica microfibers grafted with nano-titania presented antibacterial action (increase/proliferation absent) while the polysulfone-silica microfibers membrane allowed the development of *Candida albicans* colonies. Corroborating all performed antimicrobial/bacteriological analyses, we concluded that the presence of nano-titania in the composite membranes improves the behavior in terms of microorganism inhibition and absence of *Candida albicans* strain proliferation.

The complex investigation over the polysulfone-based membranes proved that the composite membranes containing silica microfibers grafted with nano-titania are characterized by mechanical properties that are comparably better than the initial polysulfone membrane and the polysulfone-silica microfibers composite membrane. The values for both tensile strain and elasticity modulus are about 14% higher compared with the polysulfone membrane.

Taking into account the determined characteristics and antimicrobial behavior when nano-titania was introduced in the polysulfone-silica microfiber membrane, we could conclude that the composite membrane PSf-SiO_2_-TiO_2_ is suitable for clinical applications. However, further investigations both in vitro and in vivo are needed for the complete assessment of the proposed membrane for GBR applications.

## Figures and Tables

**Table 1:** Permeability of polysulfone composite membranes.

Membrane Type	Water Permeability (L/m^2^·h·bar)	Ethanol Permeability (L/m^2^·h·bar)
12% PSf/NMP	43 ± 5	55 ± 6
12% PSf/NMP with SiO_2_	53 ± 6	71 ± 11
12% PSf/NMP with SiO_2_+TiO_2_	63 ± 11	97 ± 13

PSf—polysulfone; NMP—N-methyl pyrrolidone.

**Table 2:** Chemical resistance of polysulfone composite membranes.

Membrane Type	Weight Loss (%)	
	3% H_2_O_2_ in H_2_SO_4_ 2%	NaClO 5%
12% PSf/NMP	2.3 ± 0.1	3.7 ± 0.1
12% PSf/NMP with SiO_2_	1.8 ± 0.4	2.2 ± 0.4
12% PSf/NMP with SiO_2_+TiO_2_	1.4 ± 0.1	1.2 ± 0.1

PSf—polysulfone; NMP—N-methyl pyrrolidone.

**Table 3:** Bovine serum albumin (BSA) retention onto polysulfone composite membranes.

Membrane Type	Total Porosity (%)	BSA Retention (%)
12% PSf/NMP	65 ± 22	77 ± 3
12% PSf/NMP with SiO_2_	71 ± 21	78 ± 2
12% PSf/NMP with SiO_2_ + TiO_2_	74 ± 8	94 ± 5

PSf—polysulfone; NMP—N-methyl pyrrolidone.

**Table 4:** Mechanical behavior of the composite membranes.

Characteristic	Membrane Variety		
	PSf	PSf-SiO_2_ Microfibers	PSf-SiO_2_ Microfibers—TiO_2_
Max. tensile stress (MPa)	3.9 ± 0.2	3.8 ± 0.1	4.1 ± 0.1
Max. tensile strain (%)	27 ± 3	25 ± 3	31 ± 1
Elastic modulus (MPa)	89 ± 1	96 ± 1	102 ± 1
Thickness (mm)	0.426 ± 0.03	0.274 ± 0.01	0.207 ± 0.01

**Table 5:** Statistical parameters calculated for polysulfone and polysulfone composite membranes.

Characteristics	Thickness (mm)			Max. Tensile Stress (MPa)			Max. Tensile Strain (%)			Elastic Modulus (MPa)		
Statistical Parameters	PSf	PSf-SiO_2_	PSf-SiO_2_-TiO_2_	PSf	PSf-SiO_2_	PSf-SiO_2_-TiO_2_	PSf	PSf-SiO_2_	PSf-SiO_2_-TiO_2_	PSf	PSf-SiO_2_	PSf-SiO_2_-TiO_2_
**Mean**	0.426	0.274	0.207	3.87	3.78	4.09	27	25	31	89	96	102
**Standard deviation**	0.03	0.01	0.01	0.2	0.1	0.1	3	3	1	1	1	1
**Standard deviation of the mean**	0.011	0.003	0.004	0.07	0.04	0.03	1.35	1.27	0.50	0.63	0.49	0.35
**Coefficient of variation (%)**	5.89	2.37	4.04	3.83	2.05	1.55	10.97	11.39	3.60	1.59	1.17	0.77

**Table 6:** The antimicrobial action of the tested membranes determined through a disk-diffusion technique.

Microbial Strain	Zone of Inhibition—ZOI (mm)		
	PSf	PSf-SiO_2_	PSf-SiO_2_-TiO_2_
*E. coli ATCC O_125_, B_15_*	0	0	15.0 ± 0.3
*Salmonella PT 10052*	0	0	10.0 ± 0.1
*Shigella shiga*	0	0	15.0 ± 0.2
*Ps. aeruginosa ATCC 27853*	0	0	16.0 ± 0.3
*Ps. aeruginosa ATCC 15442*	0	0	10.0 ± 0.1

**Table 7:** Bacteriological analysis results for *Candida albicans* ATCC 10231 strain.

Sample Analyzed	Results	Biological Reference Interval/UM
**Polysulfone membrane**	Increase/proliferation absent	Absence of Candida albicans
**PSf-SiO_2_ membrane**	Colonies of *Candida albicans* present	Absence of Candida albicans
**PSf-SiO_2_-TiO_2_ membrane**	Increase/proliferation absent	Absence of Candida albicans
**Sabouraud Oxoid medium plate**	Present colonies of *Candida albicans*	Absence of Candida albicans
